# Kenya Hospices and Palliative Care Association: integrating palliative care in public hospitals in Kenya

**DOI:** 10.3332/ecancer.2016.655

**Published:** 2016-07-07

**Authors:** Zipporah Ali

**Affiliations:** Kenya Hospices and Palliative Care Association, PO Box 20854, 00202 Nairobi, Kenya

**Keywords:** palliative care, integration, advocacy

## Abstract

**Background:**

In Kenya, cancers as a disease group rank third as a cause of death after infectious and cardiovascular diseases. It is estimated that the annual incidence of cancer is about 37,000 new cases with an annual mortality of 28,000 cases (Kenya National Cancer Control Strategy 2010). The incidence of non-communicable diseases accounts for more than 50% of total hospital admissions and over 55% of hospital deaths (Kenya National Strategy for the Prevention and Control of Non Communicable Diseases 2015–2020). The prevalence of HIV is 6.8 (KIAS 2014). Most of these patients will benefit from palliative care services, hence the need to integrate palliative care services in the public healthcare system.

**Method:**

The process of integrating palliative care in public hospitals involved advocacy both at the national level and at the institutional level, training of healthcare professionals, and setting up services within the hospitals that we worked with. Technical support was provided to each individual institution as needed.

**Results:**

Eleven provincial hospitals across the country have now integrated palliative care services (Palliative Care Units) and are now centres of excellence. Over 220 healthcare providers have been trained, and approximately, over 30,000 patients have benefited from these services. Oral morphine is now available in the hospital palliative care units.

**Conclusion:**

As a success of the pilot project, Kenya Hospices and Palliative Care Association (KEHPCA) is now working with the Ministry of Health Kenya to integrate palliative care services in 30 other county hospitals across the country, thus ensuring more availability and access to more patients. Other developing countries can learn from Kenya’s successful experience.

## Introduction

The annual incidence of cancer in Kenya is estimated to be close to 37,000 new cases with an annual mortality of over 28,000, making cancer the third leading cause of death after infectious diseases and cardiovascular conditions. Over 60% of those affected are below the age of 70 years. In Kenya, the risk of getting cancer before the age of 75 years is 14%, while the risk of dying of cancer is estimated at 12% (National Cancer Control Strategy 2011–2016) [1]. These estimates are conservative and could be higher given that many cases go unreported and unaccounted for.

Most patients are in the advanced stages of disease and require palliative care. Even for those with early diagnosis, palliative care is important since the majority will not be able to either access or afford treatment aimed at cure. Therefore, there is a great need to expand palliative care services and bring these services close to those who need them.

While the two national referral hospitals (Kenyatta National Hospital and Moi Teaching and Referral Hospital) still remain the two main hospitals that provide close to comprehensive cancer treatment [1], the Kenya government has initiated other centres for cancer treatment. The new centres do not provide radiotherapy (this is currently only available in one public hospital- Kenyatta National Hospital). Most patients with cancer are unable to access the referral hospital due to funds, distance, and long queues. Hence, having palliative care units in other government hospitals is necessary.

Over the last 5 years Kenya Hospices and Palliative Care Association (KEHPCA) has been supporting the integration of palliative care services in 11 level 5 government hospitals [3]. The major activities undertaken over this period included the following: advocacy both at the national and at the hospital level; capacity building through training and mentorship; establishment of palliative care units through the renovation of an identified building/room and equipping them; ensuring supply of morphine and other essential palliative care medicines and providing palliative care services to patients and their families. This project has been successful and sustainable, since services are provided in government hospitals, making it accessible, affordable, and sustainable since services are maintained using hospital funds.

## Aim

The main aims of this project are to provide palliative care services for more patients and their families, thus reduce the suffering of many by improving their quality of life and creating centres of excellence in service delivery, training, and mentorship in the 11 level 5 hospitals.

## Methods

Advocacy was very instrumental as a starting point. By 2010, palliative care in Kenya was basically provided by the then few hospices existing. These were not widely spread across the country, thus making services available to only a few patients and their families. As the umbrella organisation for hospice and palliative care, Kenya Hospices and Palliative Care Association (KEHPCA) recognised the need for palliative care services to be integrated into the healthcare system in the country if more patients and families were to benefit from these services. The association approached the relevant key people at the Ministry of Health (MoH), wrote letters, and made presentations to the relevant heads of the different departments in the ministry, thus making a strong case for the need of integration. Then, inviting the minister of health to open our first and second national palliative care conferences also played a significant role in the ministry’s decision to forge a relationship with KEHPCA to integrate palliative care in government hospitals. In 2010, the director of medical services at the MoH wrote a circular to 11 provincial hospitals to ask them to work with KEHPCA to start palliative care services.

Following the circular, it now became a challenge for KEHPCA to look for funding to support the 11 hospitals to start services as the ministry did not have a budget for these new services. KEHPCA was able to source funding to support this initiative. The first activity was advocacy and creating awareness at institutional levels, and these involved several visits and meetings with the hospital management teams in each site and conducting CMEs (Continued Medical Education) to Health Care Professionals (HCPs) in each hospital. Once the concept was accepted, KEHPCA organised five-day training for 20 multidisciplinary HCPs for each hospital, which was followed by a three-day clinical placement in a nearby hospice. A total of 220 HCPs were trained over a period of one year using a curriculum developed by KEHPCA.

Each hospital selected a team leader and assigned one to two nurses to work closely with KEHPCA to set up a palliative care unit (PCU) within the hospital. Rooms were identified for the team to use; however, patients are treated within the hospital, in the wards if they are inpatients. The trained HCPs are able to identify patients who need palliative care and refer them to the PCU. The palliative care teams take part in ward rounds and are part of the decision-making teams. Once patients are discharged, the team tries to link them to a hospice, where there is one, or patients can continue to receive outpatient services at the PCU in the hospital.

One of the other challenges was getting morphine for the patients. Initially, KEHPCA had to buy morphine for the hospitals, but now the MoH does the purchasing of the morphine powder and through the Kenya Medical Supply Agency (KEMSA), the morphine is distributed to the hospital as per demand.

## Results

Eleven provincial hospitals have successfully integrated palliative care within their institutions, thus so far serving over 30,000 patients. About 220 multidisciplinary healthcare professionals were trained in the 11 hospitals; some of them have recently undertaken a higher diploma or a degree training in palliative care, thus strengthening the hospitals’ capacity to become centres of excellence in service provision and training. The 11 hospitals are now used as clinical and mentorship sites for district (now county) and other hospitals which want to integrate palliative care.

The Kenya Medical Training College, the biggest training institution in Kenya, has identified these hospitals as clinical placement sites for their students undertaking the higher diploma course in palliative care. The project is now being extended to 30 other county hospitals across the country. The trained palliative care teams in these hospitals provide supervision and mentorship of other trainees.

By integrating palliative care in the public hospitals (where the majority of patients are seen), KEHPCA, in partnership with the Ministry of Health, through the service delivery models that have been developed as a comprehensive and public health approach, has been able to reduce the suffering of many patients with cancer.

Other initiatives that have indirectly resulted in this programme to improve palliative care for both adults and children in Kenya include the following:

- recognising palliative care as a basic right in The Kenya National Patients’ Rights Charter which states that *‘every person, patient or client has a right to access health care which shall include promotive, preventive, curative, reproductive, rehabilitative, and palliative care’*
- National Palliative Care Guidelines [7]
- integrating palliative care education in undergraduate medical and nursing curriculum
- integrating palliative care in the National Cancer Control Strategy, the National Guidelines for Cancer Management,
- including palliative care in the Kenya National Strategy for the Prevention and Control of Non-Communicable Diseases 2015–2020 and the community health volunteer (CHVs) non-communicable diseases training manual
- advocating to the Ministry of Health to procure morphine powder for constitution of oral morphine for government hospitals that have integrated palliative care
- developing a higher diploma course for nurses at the Kenya Medical Training College, thus making it possible to have specialist nurses in palliative care who are expected to be both trainers and mentors of other healthcare professionals.

## Conclusion

For palliative care to be accessible in a developing country, a public health approach is important. Integrating palliative care into the health care system improves access, affordability, and holds the government responsible.

However, there is still a lot that needs to be done to ensure good coverage of palliative care services across the country. All healthcare providers need some training in the basic principals in palliative care as they will all encounter a patient in need. The government needs to budget for palliative care services and work with other stakeholders and partners to make it available at all levels of care, including at the community level. Counties need to include palliative care in their local health strategies and plans.
